# Human Leukocyte Antigen (HLA) Class I Susceptible Alleles Against COVID-19 Increase Both Infection and Severity Rate

**DOI:** 10.7759/cureus.12239

**Published:** 2020-12-23

**Authors:** Tomoo Ishii

**Affiliations:** 1 Orthopedic Surgery, Tokyo Medical University Ibaraki Medical Center, Ibaraki, JPN

**Keywords:** covid-19, sars-cov-2, hla class i, hla-a, hla-b, hla-a/b haplotype, cytotoxic t lymphocyte (ctl), infection rate, severity rate

## Abstract

Introduction

Each country's difference in the severity rate of severe acute respiratory syndrome coronavirus 2 (SARS-CoV-2) may be explained by the difference in human leukocyte antigen (HLA) class I molecules, which affects the reactivity of cytotoxic T lymphocyte (CTL).

Methods

To clarify the relationship between HLA class I and the severity rate, the binding repertoires of each HLA class I allele to SARS-CoV-2 peptides and the allele frequencies of HLA-A, -B, and -A/B haplotypes in each country were quoted.

Results

HLA-A1 and the number of deaths per million population (severity rate) in each country had an exponential approximation correlation with correlation coefficient R=0.4879. In addition, the correlation between the infected cases per million (infection rate) and the severity rate was linearly approximated, with R=0.7422. Weak HLA-A alleles with a repertoire of under 300 also had an exponential approximation correlation with the severity rate (R=0.5972), whereas there was a linear approximation with the infection rate (R=0.6808). Weak HLA-B alleles of 30 repertoires or less had no correlation with the severity rate (R=-0.1530). The weak HLA-A/B haplotype has a stronger effect on the severity rate than the weak HLA-A alone. Therefore, the simple HLA class I susceptibility index was calculated, and a strong correlation (R=0.7388) of an exponential approximation with the severity rate was obtained.

Conclusions

HLA class I susceptible alleles against COVID-19 increase both infection and severity rate. The weak HLA-A is a major factor of severity rate, whereas the weak -B alone has no correlation. However, the weak HLA-A/B haplotype has a stronger effect on the severity rate than the weak -A alone.

## Introduction

In viral infections, human leukocyte antigen (HLA) class I plays an important role in their severity and progression. With the severe acute respiratory syndrome coronavirus 2 (SARS-CoV-1) virus, Taiwanese researchers have shown HLA-B46 to cause severe symptoms [1]. In human immunodeficiency virus (HIV) epitopes, HLA class I molecules show different reactivity of cytotoxic T lymphocyte (CTL), and it is known that HLA with low reactivity poses a high risk of acquired immunodeficiency syndrome (AIDS) becoming severe [2]. The mechanism by which class I molecules, such as HLA-A, -B, and -C, are involved in the aggravation of viral infections is the viral antigen-presenting part of CTL in cell-mediated immunity. HLA class I carries antigen-presenting peptides (viral epitopes) and activates CTL. The number of binding repertoires of fractionated viral epitopes differ greatly depending on the type of virus and each HLA type and are factors that determine the immunocompetence of CTL. The author speculated that the differences in HLA class I molecules may explain the high severity of the SARS-CoV-2 virus in Europe and the United States and the low severity of the disease in East Asia.

## Materials and methods

It is difficult to clarify the rate of serious cases infected by SARS-CoV-2 virus in each country, and as a hypothesis, in a country where the number of infected cases has spread to some extent, the number of deaths per million population is considered to correlate well with the rate of serious cases (severity rate).

Forty-three countries were targeted in the northern hemisphere within the first 50 places in the world's nominal gross domestic product (GDP) ranking in 2018 to unify the natural environmental conditions of each country. As of data on May 14, 2020, the author calculated the number of deaths and infected cases per million population by the latest population statistics in each country. Countries under 100 infected cases per million population (Vietnam, Taiwan, Nigeria, Thailand, India, China) were also excluded in the research to judge that degree of infection has not progressed to some extent in those countries.

Looking at the frequencies of HLA class I molecules in Europe, North America, and East Asia, the biggest difference was the HLA-A1 antigen. The frequency of HLA-A1 in each country was investigated from the "Allele Frequency Net Database" (http://www.allelefrequencies.net/hla.asp). The frequency of the HLA-A*01 allele was examined, but in many countries, the frequency of multiple studies was published and the sample with the largest number and universality was used as the representative value of the country. The frequency of the United States was listed by race, but Caucasian, which has the largest number of samples and is the largest population, was the representative value. There are three countries (Canada, Denmark, Egypt) where the frequency of the HLA-A * 01 allele does not appear. Excluding them, there are a total of 34 countries (Table 1).

**Table 1:** Frequency of HLA-A * 01 allele and deaths and infected cases per million population in each country

	Total deaths	Infected cases	Population	A*01 allele %	Deaths per million population	Infected cases per million population
Belgium	8,843	53,981	11,492,000	15.50	769.49	4697.27
Spain	27,321	229,540	46,930,000	10.25	582.16	4891.11
Italy	33,106	222,104	60,600,000	10.50	546.30	3665.08
United kingdom	33,614	233,151	66,000,000	19.06	509.30	3532.59
France	27,425	141,356	66,990,000	13.20	409.39	2110.11
Sweden	3,460	27,909	9,838,000	13.60	351.70	2836.86
Netherland	5,590	43,481	17,380,000	17.50	321.63	2501.78
Ireland	1,497	23,401	4,920,000	23.59	304.27	4756.30
United States	85,813	1,417,350	327,750,000	16.48	261.82	4324.49
Switzerland	1,563	30,330	8,540,000	10.02	183.02	3551.52
Portugal	1,184	28,319	10,372,000	10.70	114.15	2730.33
Germany	8,015	175,498	83,150,000	15.14	96.39	2110.62
Iran	6,854	114,533	80,000,000	11.60	85.68	1431.66
Austria	624	15,964	8,794,000	14.50	70.96	1815.33
Finland	284	6,054	5,503,000	8.90	51.61	1100.13
Romania	1,016	16,002	19,760,000	12.00	51.42	809.82
Turkey	4,007	144,749	79,512,000	10.46	50.39	1820.47
Norway	229	8,158	5,368,000	15.92	42.66	1519.75
Mexico	3,926	38,324	126,190,000	6.06	31.11	303.70
Israel	262	16,539	8,880,000	15.20	29.50	1862.50
Czech Republic	290	8,269	10,650,000	16.11	27.23	776.43
Poland	861	17,204	38,224,000	13.98	22.53	450.08
United Arab Emirates	208	21,084	9,630,000	6.20	21.60	2189.41
Russia	2,305	252,245	143,965,000	11.40	16.01	1752.13
Colombia	493	12,272	49,650,000	6.35	9.93	247.17
Saudi Arabia	283	46,869	32,276,000	7.01	8.77	1452.13
Phillippines	772	11,618	109,800,000	0.00	7.03	105.81
Japan	687	16,079	126,170,000	0.40	5.45	127.44
South Korea	260	10,991	51,270,000	1.90	5.07	214.37
Singapore	21	25,346	5,640,000	3.20	3.72	4493.97
Malaysia	111	6,779	31,187,000	2.10	3.56	217.37
Pakistan	770	35,778	277,770,000	6.30	2.77	128.80
Bangladesh	283	18,863	163,650,000	9.50	1.73	115.26
Hong Kong	4	1,050	3,980,000	0.84	1.01	263.82

To clarify the relationship between HLA class I and severity rate, the author downloaded raw data from a theoretical calculation paper of the binding repertoires of each HLA class I allele to SARS-CoV-2 peptides [3]. The alleles of a low number of binding repertoires in the HLA-A, -B, and -A/B haplotypes were investigated for the correlation between allele frequencies and severity rates in each country.

## Results

**Figure 1:**
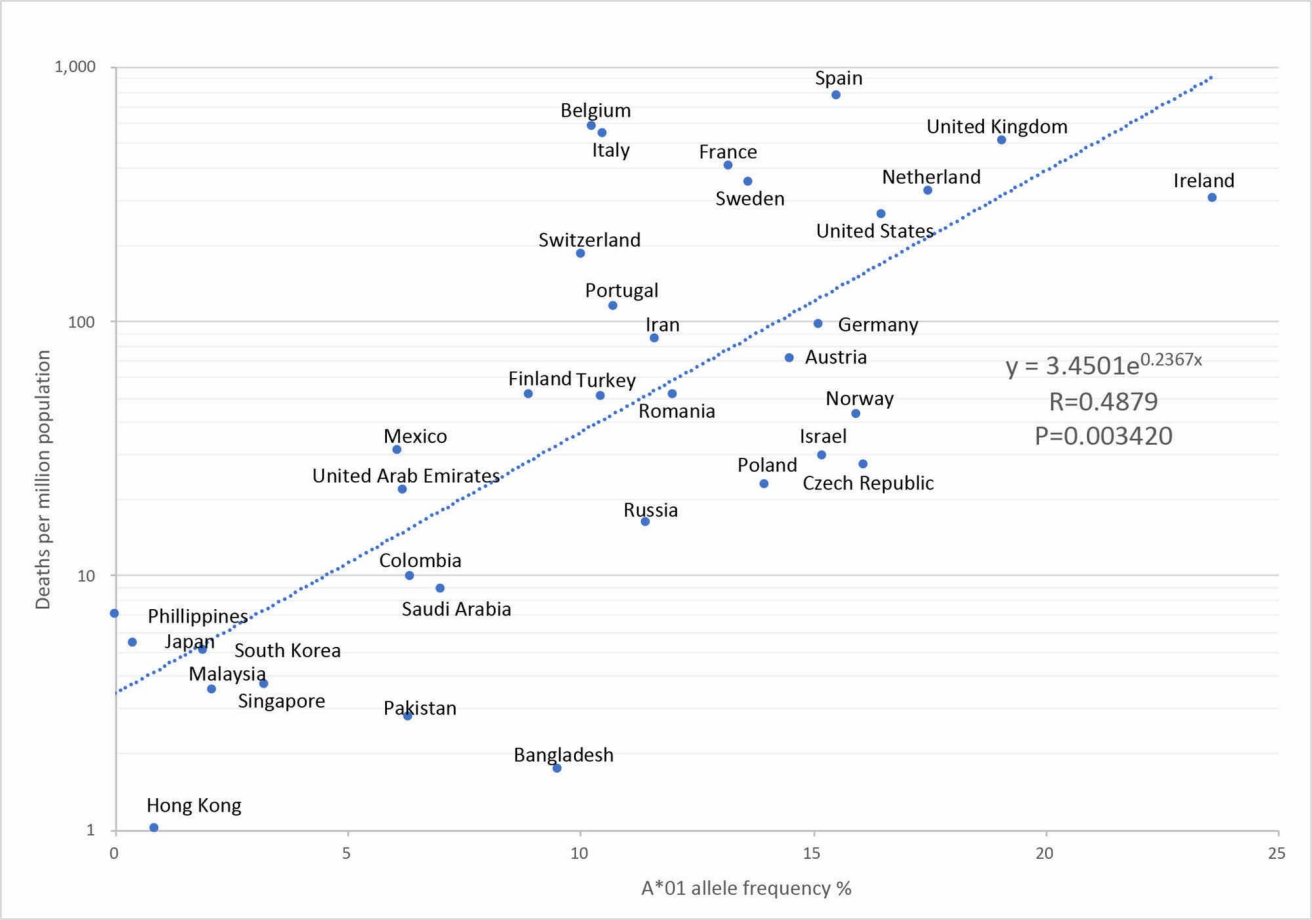
Correlation between the A*01 allele and severity rate

The correlation coefficient R=0.4879 and P=0.0034 between HLA-A1 and the number of deaths per million were found to be moderate and significant in an exponential approximation correlation (Figure 1).

**Figure 2:**
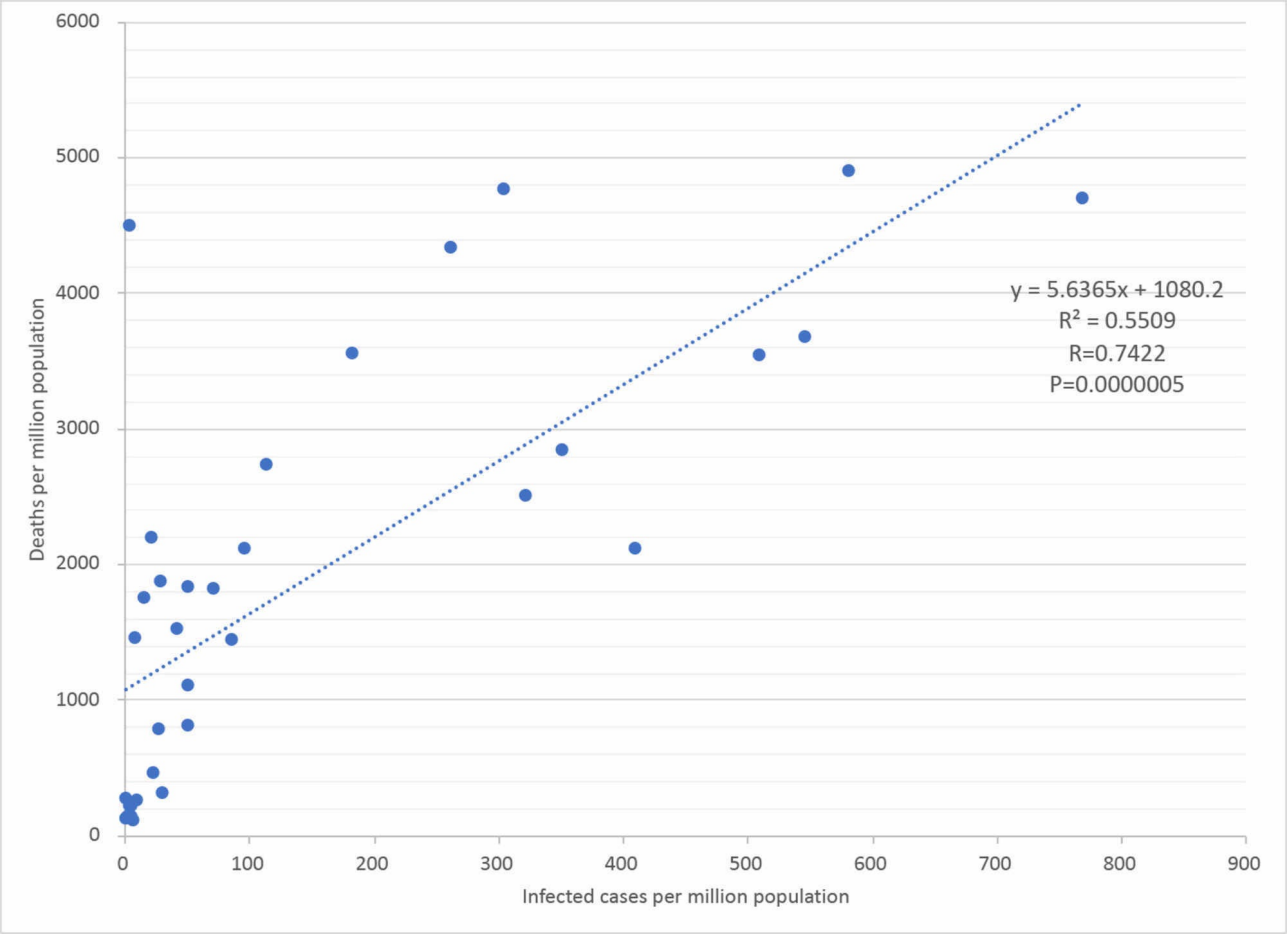
Correlation between infected cases and deaths per million population

In addition, the correlation between the infected cases (infection rate) and the deaths (severity rate) per million was linearly approximated, showing a strong positive correlation with R=0.7422 (Figure 2). It shows that the higher the infection rate, the higher the severity rate.

The number of binding repertoires of HLA-A1 is 183 in HLA-A01: 01 and 126 in HLA-A01: 02, and the average of all HLA-A alleles is 498, so the binding repertoire of HLA-A1 is small. The frequencies of binding repertoires of HLA-A alleles under 300 were counted and the correlation with deaths per million population in each country calculated. There are nine countries (Sweden, Netherland, Turkey, Norway, Israel, Singapore, Malaysia, Pakistan, Bangladesh) where the frequency of HLA-A alleles also does not appear. Excluding them, there are a total of 25 countries (Table 2).

**Table 2:** Frequencies of HLA-A alleles on the number of repertoires under 300 of SARS-CoV-2 peptides

Allele under 300	A*01:01%	A*01:02%	A*02:07%	A*24:07%	A*25:01%	A*26:01%	A*26:03%	A*30:04%	A*32:01%	A*33:01%	A*34:01%	A*36:01%	A*66:01%	A*66:02%	A*74:01%	A*80:01%	Total frequency %
Belgium	24.6	0	0	0	3.6	3	0	0	3.7	0.5	0	0	0.8	0	0	0	36.2
Spain	9.85	0.29	0	0.01	1.48	4.23	0	0.32	3.84	1.91	0	0.03	0.73	0.01	0.06	0.13	22.89
Italy	10.2	0.3	0.1	0.2	1.7	4.1	0	0.7	3.7	2	0	0.1	0.8	0	0.1	0	24
United Kingdom	20.62	0	0.1	0.1	0.77	1.93	0	0.19	2.9	0.3	0	0	0.29	0	0.1	0.05	27.35
France	15	0	0	0	0.8	4.3	0	0.4	7.2	2.3	0	0	0.4	0	0	0	30.4
Ireland	25	0	0	0	3.6	3	0	0	4.4	0.4	0	0	0.8	0	0	0	37.2
United States	16.46	0.01	0.00392	0.0069	2.1	3.09	0.0036	0.15	3.55	0.81	0.00398	0.01	0.41	0.00276	0.04	0.01	26.66116
Switzerland	10.02	0	0	0	2	2.43	0.11	0	4.36	3.41	0	0	0.85	0			23.18
Portugal	5.9	0.8	0	0	0.5	3.5	0	0	4.9	3	0	0.3	1.1	0	0	0	20
Germany	15.07	0.009	0.04	0.03	2.38	3.61	0.001	0.16	3.64	0.81	0.009	0.001	0	0.004	0.1	0.02	25.884
Iran	3.1	4.7	0	0	1.6	4.7	0	0	2.3	3.1	0	0	0	0	0		19.5
Austria	14.6	0	0	0	2.2	4.2	0	0.2	2.8	0.5	0	0	0.2	0	0	0	24.7
Finland	8.9	0	0	0	1.1	1.1	0	0	2.2	0	0	0	0	0	0	0	13.3
Romania	12.2	0	0	0	3.5	3.8	0.3	0.1	5	1	0	0	0.1	0	0	0	26
Mexico	6.06	0	0	0	0.91	2.88	0	0	2.4	1.2	0	0	0.3	0	0.3	0	14.05
Czech Republic	16.7	0.01	0.04	0.03	4.02	5.03	0	0.13	3.18	0.9	0.01	0	0.82	0	0	0.02	30.89
Poland	13.72	0.0064	0.05	0.03	5.2	4.86	0	0	2.8	1	0	0	0.72	0	0.0085	0	28.3949
United Arab Emirates	6.73	0	0	0	0	1.92	0	0.96	9.62	0.96	0.96	0	0.96	0	3.85	0	25.96
Russia	10.14	0	0.05	0	4.61	4.79	0.05	0.05	2.61	0.94	0	0	0.41	0	0	0	23.65
Colombia	6.08	0.27	0	0	0.03	0.89	2.05	0.27	2.39	1.67	0	0.07	0.41	0.21	0.58	0.07	14.99
Saudi Arabia	11.5	0	0	0.32	0	3.5	0	1.2	2.4	1.2	0.32	0	1.2	0	0.5	0	22.14
Phillippines	0	0	0	9	0	0	0	0	0	0	13	0	0	0	0	0	22
Japan	0.4	0	3.46	0.02	0	7.35	2.29	0.02	0.02	0.01	0.01	0	0.005	0	0	0.5	14.085
South Korea	1.95	0.01	3.4	0	0	3.84	0.86	0	0.61	0	0	0	0	0	0	0	10.67
Hong Kong	0.11	0	13.1	0.51	0	1.7	0	0	0.39	0	0.3	0	0.1	0	0.3	0	16.51

**Figure 3:**
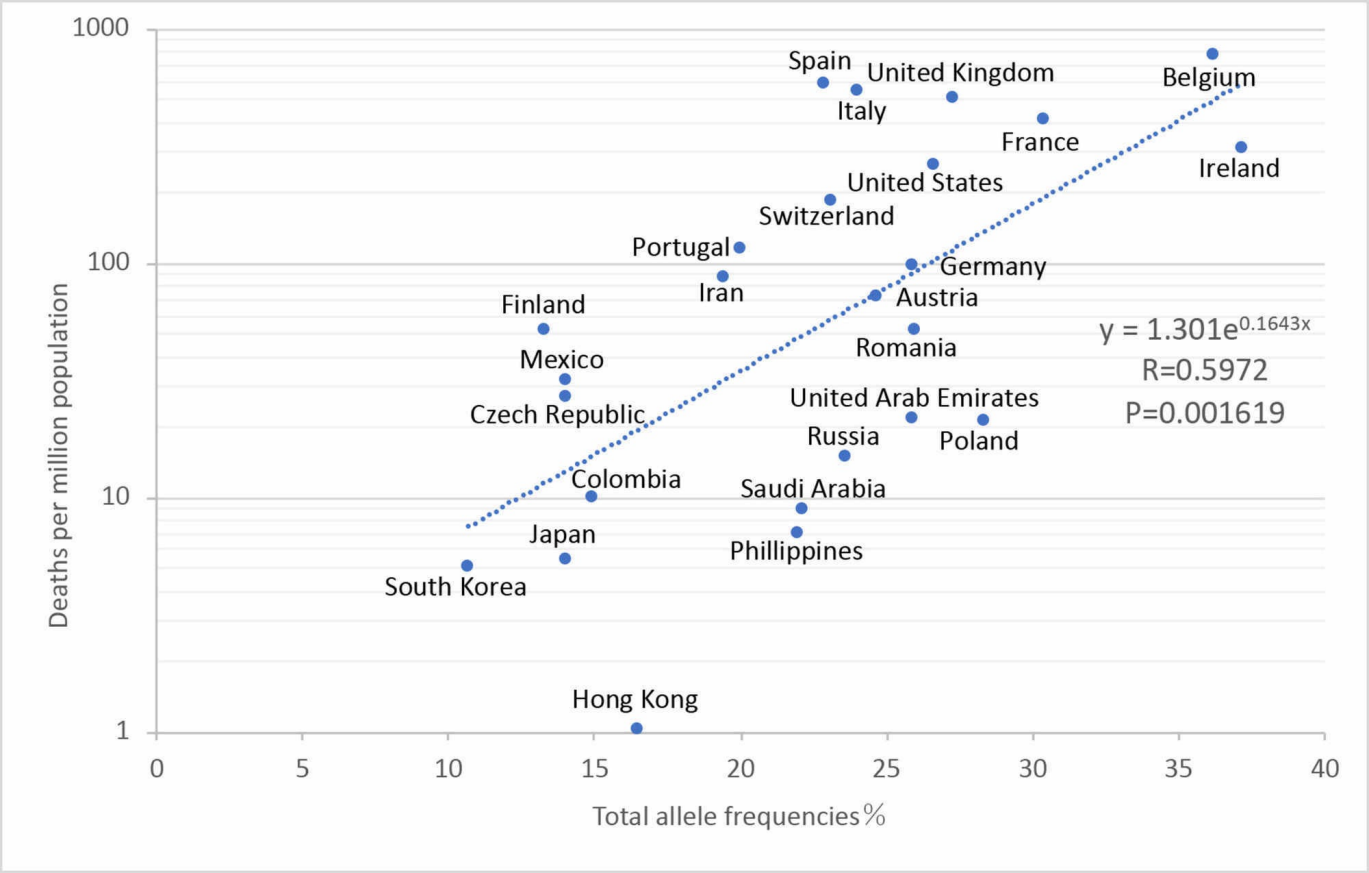
Correlation between total allele frequencies under 300 repertoires of SARS-CoV-2 peptides and deaths per million population

In HLA-A, alleles with a combined repertoire of under 300 and the deaths per million are in an exponential approximated correlation, with the correlation coefficient R=0.5972 (P=0.001619), increasing as compared to HLA-A1 (Figure 3). The severity rate is found to exponentially correlate with a decrease in the number of repertoires binding to SARS-CoV-2 viral peptides. Correlations were observed at a frequency of HLA-A with a binding repertoire number under 200, but a correlation coefficient under 300 was better.

**Figure 4:**
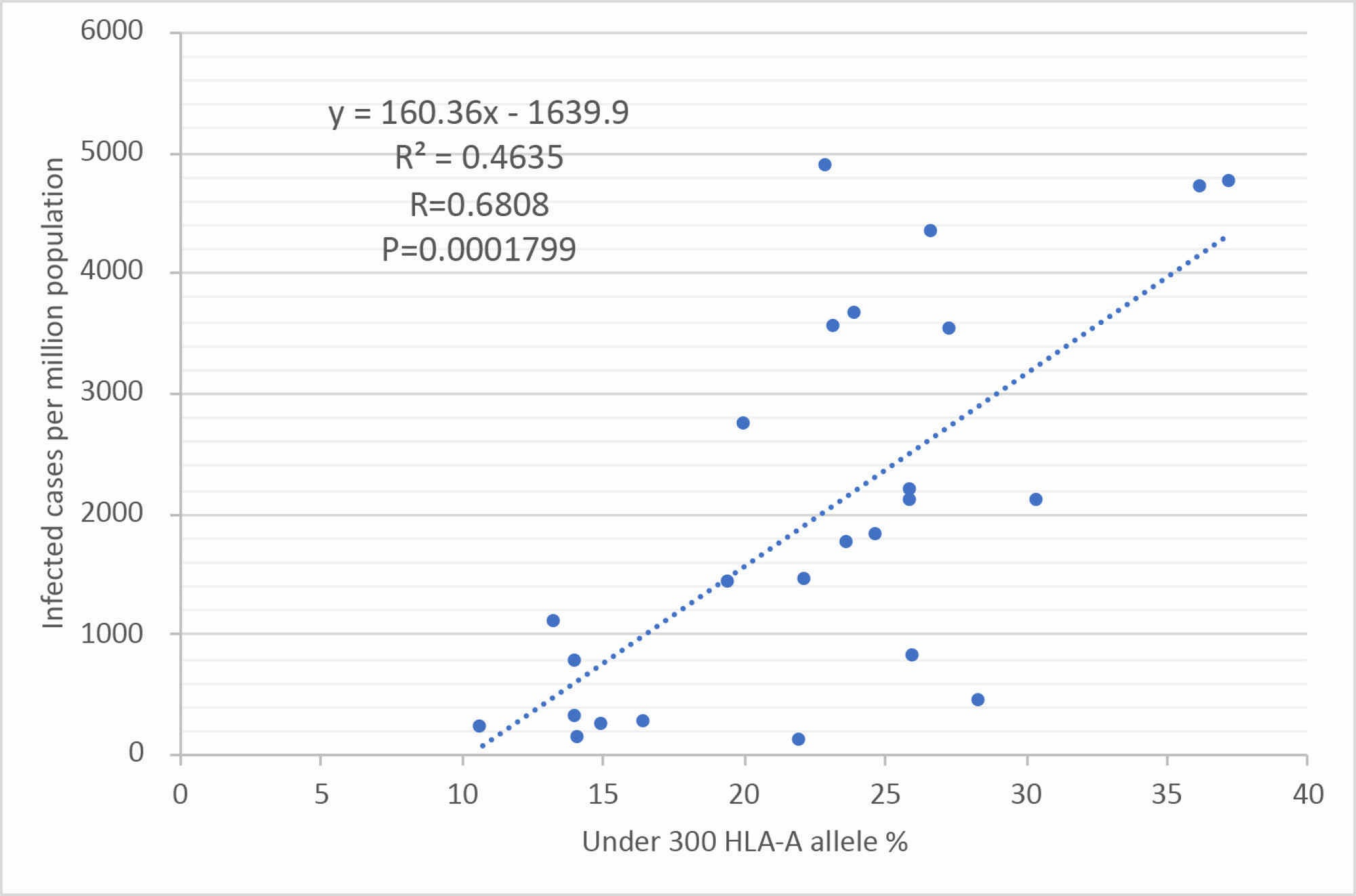
Correlation between total allele frequencies under 300 repertoires of SARS-CoV-2 peptides and infected cases per million population

Looking at the correlation between the HLA-A allele under 300 and infected cases per million population, a linear approximation was made, and the correlation coefficient (R=0.6808) was higher than the deaths per million population (Figure 4). The infection rate is found to linearly correlate with repertoires under 300 binding to HLA-A SARS-CoV-2 viral peptides.

The number of HLA-B binding repertoires SARS-CoV-2 peptides was low, with an average of 209 and a median value of 125. There were 17 genes of 30 repertoires or less, and the least allele was B46: 01 at three repertoires. The total frequency of 17 alleles with 30 repertoires or less was examined for whether the frequency correlates with the number of deaths per million population (Table 3), but there was no correlation with the coefficient R=-0.1530. Repertoires under 20 or 50 also had no correlation. HLA-B alone does not have a direct correlation.

**Table 3:** Frequencies of HLA-B alleles on the number of repertoires of 30 or less SARS-CoV-2 peptides

Allele 30 or less	B13:02	B14:01	B14:02	B14:03	B15:10	B27:03	B35:03	B37:01	B40:12	B44:05	B46:01	B48:01	B48:03	B51:08	B52:01	B58:02	B82:01	Total frequency％
Spain	1.52	1.85	3.99	0.03	0.13	0.05	2.23	1.07	0	0.29	0	0.07	0	0.25	1.4	0	0	12.88
Italy	0	1.2	5.7	0	0.2	0	3.8	1.2	0	0.6	0	0	0	0.2	1.2	0	0	14.1
United Kingdom	2.31	1.16	2.6	0	0	0.1	0.48	2.12	0	0.29	0.1	0	0	0	1.06	0	0	10.22
Ireland	1.2	2.4	2.4	0	0	0	1	1.2	0	0	0	0	0	0	0.6	0	0	8.8
United States	2.39	1.02	2.86	0.00141	0.04	0.0068	1.62	1.44	0.00028	0.29	0.00752	0.07	0.00028	0.1	2	0.01	0.0004	11.85669
Switzerland	2.27	1.07	1.49	0	0	0	4.52	1.08	0	0.34	0	0	0	0.4	1.81	0.13	0	13.11
Portgul	1	2	3.1	0	0	0	1	0	0	0	0	0	0	0	0	0	0	7.1
Germany	3.47	0.49	1.98	0.005	0.02	0.01	2.46	1.34	0.001	0.62	0.04	0.07	0	0.15	1.13	0.01	0.001	11.797
Iran	0.5	0	1.5	0	1	0	1	0	0	0	0	0	0	0.5	2.5	1	0	8
Austria	4.8	0.5	2.2	0	0	0	3.5	1	0	0.2	0	0	0	0	1.5	0	0	13.7
Romania	4.1	0.6	1.7	0	0.1	3.2	0.9	0.9	0	0	0.1	0	0	0	3.2	0	0	14.8
Mexico	0.15	1,82	3.33	0	0	0	0	0.91	0	0	0.15	3.94	0	0	1.21	0	0	9.69
Czech Republic	4.75	0.41	1.65	0	0	0.02	3.15	1.05	0	0.66	0.04	0.06	0	0.21	1.17	0	0	13.17
Poland	5.45	0.35	0.35	1.41	0.007	0.02	3.4	1.1	0	0.85	0.05	0.16	0	0.08	1.54	0	0	14.767
United Arab Emirates	0	0	2.88	0	0	0	1.92	0	0	0.96	0	0	0	0	5.77	2.88	0	14.41
Russia	4.92	0.19	1.73	0	0	0	2.53	0.7	0	0.42	0	0.61	0	0.09	1.73	0	0	12.92
Colombia	0.92	0.89	4	0.03	0.65	0.03	1.2	0.41	0	0.14	0	0.147	0	0.41	1.47	0.55	0	10.847
Saudi Arabia	0.01	0	1.5	0	0.2	0.5	1.7	2	0	0	0	0	0	0.7	1.2	0	0	7.81
Phillippines	0	0	0	0	0	0	0	0	0	0	0	8	0	0	0	0	0	8
Japan	0.29	0.02	0.01	0	0	0	0	0.5	0	0	4.77	2.88	0	0	11.09	0	0	19.56
South Korea	3.09	1.24	0.02	0	0	0	0.52	1.47	0	0	4.74	3.56	0	0	2.98	0	0	17.62
Hong Kong	1.72	0	0.02	0	0.02	0.16	0.83	0.39	0	0	14.85	1.16	0.69	0.0067	0.76	0	0	20.6067

If the HLA-A allele alone is associated with the severity rate in HLA class I, it cannot explain the facts that Africans had a high severity rate in the U.S. and the United Kingdom. The frequency in the United States in this study is represented by Caucasians, which is the largest population, and the total frequency of the HLA-A allele under 300 was 26.66%, but the frequency of Africans was rather low, at 21.76%. As a hypothesis, it is considered that the HLA-A/B haplotype of low binding sites in each plays a more important role in the severity rate. The frequencies of haplotypes determined that the HLA-A alleles (under 300) and HLA-B (30 or less) of Caucasians and Africans were 0.788 and 7.41%, respectively (Table 4 and Table 5).

**Table 4:** Frequencies of weak haplotypes determined HLA-A alleles (under 300) and HLA-B (30 or less) of Caucasians in the United States

Allele Caucasian	A*01:01	A*01:02	A*02:07	A*24:07	A*25:01	A*26:01	A*26:03	A*30:04	A*32:01	A*33:01	A*34:01	A*36:01	A*66:01	A*66:02	A*74:01	A*80:01	Total freuency％
HLA-B13:02	0	0	0	0	0	0	0	0	0	0	0	0	0	0	0	0	0
HLA-B14:01	0	0	0	0	0	0	0	0.0017	0	0	0	0	0	0	0	0	0.0017
HLA-B14:02	0	0	0	0	0	0	0	0	0	0.4423	0	0	0	0	0	0	0.4423
HLA-B14:03	0	0	0	0	0	0	0	0	0	0	0	0	0	0	0	0	0
HLA-B15:10	0	0	0	0	0	0	0	0	0	0	0	0	0	0	0	0	0
HLA-B27:03	0	0	0	0	0	0	0	0	0	0	0	0	0	0	0	0	0
HLA-B35:03	0	0	0	0	0	0	0	0	0	0	0	0	0	0	0	0	0
HLA-B37:01	0.159	0	0	0	0	0	0	0	0	0	0	0	0	0	0	0	0.159
HLA-B40:12	0	0	0	0	0	0	0	0	0	0	0	0	0	0	0	0	0
HLA-B44:05	0	0	0	0	0	0	0	0	0	0	0	0	0	0	0	0	0
HLA-B46:01	0	0	0.0008595	0	0	0	0	0	0	0	0	0	0	0	0	0	0.0008595
HLA-B48:01	0	0	0	0	0	0	0	0	0	0	0	0	0	0	0	0	0
HLA-B48:03	0	0	0	0	0	0	0	0	0	0	0	0	0	0	0	0	0
HLA-B51:08	0	0	0	0	0	0	0	0	0	0	0	0	0	0	0	0	0
HLA-B52:01	0.1846	0	0	0	0	0	0	0	0	0	0	0	0	0	0	0	0.1846
HLA-B58:02	0	0	0	0	0	0	0	0	0	0	0	0	0	0	0	0	0
HLA-B82:01	0	0	0	0	0	0	0	0	0	0	0	0	0	0	0	0	0
Total freuency％	0.3436	0	0.0008595	0	0	0	0	0.0017	0	0.4423	0	0	0	0	0	0	0.7884595

**Table 5:** Frequencies of weak haplotypes determined HLA-A alleles (under 300) and HLA-B (30 or less) of Africans in the United States

Allele African	A*01:01	A*01:02	A*02:07	A*24:07	A*25:01	A*26:01	A*26:03	A*30:04	A*32:01	A*33:01	A*34:01	A*36:01	A*66:01	A*66:02	A*74:01	A*80:01	Total freuency％
HLA-B13:02	0.039	0	0	0	0	0.022	0	0	0	0.065	0.021	0	0.048	0	0.19	0	0.385
HLA-B14:01	0.028	0	0	0	0	0	0	0	0.299	0.044	0	0	0.021	0	0.086	0	0.478
HLA-B14:02	0	0	0	0	0	0.046	0	0	0.064	0.675	0	0.112	0	0	0.43	0	1.327
HLA-B14:03	0	0	0	0	0	0	0	0	0	0.063	0	0.044	0	0	0	0	0.107
HLA-B15:10	0.308	0	0	0	0	0.021	0	0	0	0.09	0	0.062	0.022	0.072	0.205	0	0.78
HLA-B27:03	0.129	0	0	0	0	0	0	0	0	0.02	0	0	0	0	0	0	0.149
HLA-B35:03	0.047	0	0	0	0	0	0	0	0	0	0	0.065	0	0	0	0	0.112
HLA-B37:01	0.389	0	0	0	0.01	0.064	0	0.043	0.065	0	0	0.021	0	0	0	0	0.592
HLA-B40:12	0	0	0	0	0	0	0	0	0	0	0	0	0	0.022	0	0	0.022
HLA-B44:05	0.059	0	0	0	0	0	0	0	0	0	0	0	0	0	0	0	0.059
HLA-B46:01	0	0	0	0	0	0	0	0	0	0	0	0	0	0	0	0	0
HLA-B48:01	0	0	0	0	0	0	0	0	0	0	0	0	0	0	0	0	0
HLA-B48:03	0	0	0		0	0	0	0	0	0	0	0	0	0	0	0	0
HLA-B51:08	0	0	0	0	0	0	0	0	0	0	0	0	0	0	0	0	0
HLA-B52:01	0.305	0	0	0	0	0.044	0	0	0.044	0	0	0.137	0.021	0	0.113	0.065	0.729
HLA-B58:02	0.15	0	0	0	0	0.214	0	0	0.074	0.022	0	0.16	0.753	0	0.98	0.061	2.414
HLA-B82:01	0.086	0	0	0	0	0	0	0.042	0	0.022	0	0.043	0	0	0.022	0.043	0.258
Total freuency％	1.54	0	0	0	0.01	0.411	0	0.085	0.546	1.001	0.021	0.644	0.865	0.094	2.026	0.169	7.412

Africans were 9.4 times on the frequency of the haplotypes as compared to Caucasians, so these haplotypes may work synergistically stronger on severity than HLA-A under 300 alone.

In fact, each weak HLA-A has its own susceptibility index, and so would the weak HLA-A/B haplotype. It is assumed that the integral value of those indices correlates best with the severity rate. Here, the total frequency of HLA-A alleles with a repertoire number of under 300 is the first index. The frequency of the weak HLA-A/B haplotype composed by HLA-A under 300 and HLA-B of 30 or less is the second index. Assuming that the weak haplotypes act synergistically, the value obtained by multiplying the second index by the coefficient and the first index was considered the HLA class I susceptibility index. 

The weak haplotype frequencies in each country were obtained from 11 countries (Table 6). In the weak HLA-A/B haplotype, Italy had an outstanding high of 15.38%.

**Table 6:** Frequencies of weak HLA-A alleles, weak HLA-A/B haplotype, and weak HLA-A +1.2 times weak haplotype (HLA class I susceptibility index)

	Weak HLA-A under 300 repertoires ％	Weak HLA-A (under 300) and B(30 or less）haplotype %	Weak HLA-A (under 300) +1.2times weak haplotype % (HLA class I susceptible index)	Deaths per million population
Spain	22.89	5.29	29.238	582.16
Italy	24	15.38	42.456	546.30
United Kingdom	27.35	3.7431	31.84172	509.30
United States	26.66116	0.78846	27.607312	261.82
Mexico	14.05	1.818	16.2316	31.11
Poland	28.3949	2.7805	31.7315	22.53
Russia	23.65	2.4376	26.57512	16.01
Colombia	14.99	2.8674	18.43088	9.93
Japan	14.085	3.9204	18.78948	5.45
South Korea	10.67	9.6	22.19	5.07
Hong Kong	16.51	0.7153	17.36836	1.01

**Figure 5:**
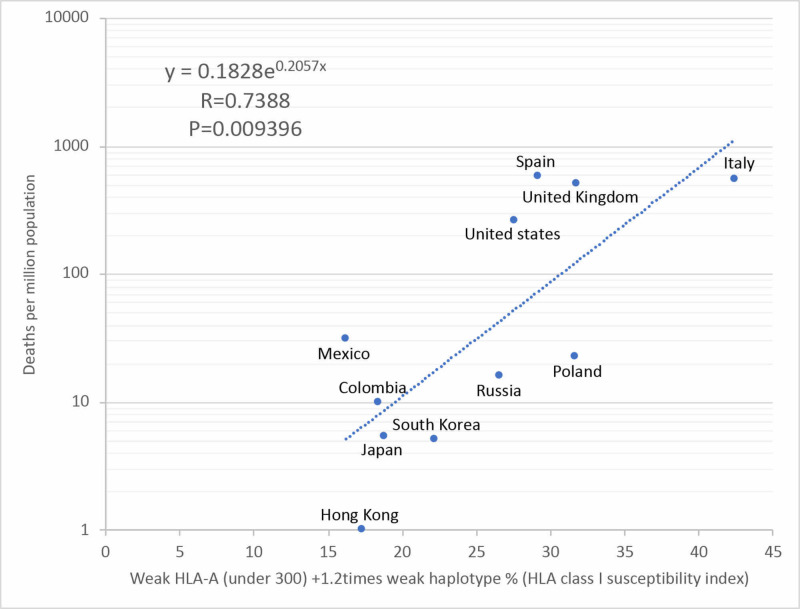
Correlation between total frequency of weak HLA-A (under 300) +1.2 times weak haplotype on SARS-CoV-2 peptides (HLA class I susceptibility index) and deaths per million population in each country

In the HLA class I susceptibility index, when the value with the highest correlation coefficient was calculated, the coefficient was 1.2. The HLA class I susceptibility index of each country and the number of deaths per million population were in an exponential approximate correlation, and the correlation coefficient shows a strong correlation with R=0.7388 (P=0.009396), and the exponential regression equation of y = 0.1828e^0.2057x^ was obtained (Figure 5). When the estimated number of deaths per million Americans in the United States was calculated from the exponential regression equation obtained here, African deaths were 1.87 times that of Caucasians. HLA class I was involved as one of the causes of the high severity rate of Africans in the United States.

## Discussion

Viruses are pathogens that enter cells and proliferate, and CTL, which recognizes fragmented viral epitopes, plays an important role in disease regulation. In HLA class I, HLA-A alleles were the major susceptibility and severity factor as compared to -B in this study. The average number of repertoires in HLA-A against SARS-CoV-2 peptides is 2.5 times higher than that of -B, so the role of CTL activity is correspondingly large, which may be a major reason.

In a report on the SARS-CoV-1 virus in Taiwan [1], HLA-B46:01 has a large number of infected people and significantly more severely ill patients (ventilator-wearers and fatalities). HLA-B46:01 is also the SARS-CoV-2 allele with the lowest number of binding repertoires [3], but HLA-B alone was not directly correlated to severity in this study. Considering the report from Taiwan, the HLA-B46:01 frequency was 16 (21.6%) in the 74 alleles with 37 cases of SARS-CoV-1 infection and suspected infection, but the frequency in HLA-B46:01 was originally 18.2%. Unlike the content of the paper, no significant difference in infection rate was apparent. However, five of the six patients with severe disease had a significantly higher allele frequency. Looking at HLA-A in those patients, the A24:02 allele was 21.6%, which had a relatively low repertoire number of 329 in SARS-CoV-2 peptides. HLA-A24:02 was positive in four out of six patients with severe illness and three out of four cases had a haplotype with HLA-B46:01. Since the frequency of the haplotype is 1.1%, it is considered that this HLA-A/B haplotype is most associated with aggravation.

In the weak HLA-A/B haplotype, Italy had an outstanding high of 15.38%. Italy announced all deaths in the country during the two months of March and April 2020 to the average death toll of the same period over the past five years at around 47,000, and 19,000 uncounted deaths may be caused by COVID-19. In this way, the haplotype with weak HLA-A/B affects the severity rate more strongly than weak HLA-A alone.

Environmental factors, such as medical environment, living environment, and lifestyle, and political measures against infection also increase the number of infected people. However, although these factors may increase the number of deaths by increasing the number of infected people, they are not the factors that increase the severity rate in infection cases. The World Health Organization (WHO) has pointed out that individual factors that raise the severity rate include 65 years and older, living in a nursing home or long-term care facility, chronic lung disease, moderate to severe asthma, serious heart conditions, immunocompromised people, severe obesity, diabetes, chronic kidney disease undergoing dialysis, and liver disease. Most of these are due to increased ACEII receptor expression and decreased innate or acquired immunity.

A significant correlation between weak HLA class I and the number of deaths per million population among many risk factors of severity indicates that HLA class I is a major factor in the severity of COVID-19. Although it takes several days for CTL acquisition immunity, SARS-CoV-2 shares many epitopes with other coronaviruses, and, theoretically, there is cross-protective immunity [3]. For this reason, HLA class I acts as the acquired immunity of CTL from an early stage and would suppress the onset of infection, which leads to a decrease in the infection rate. In this study, both the weak HLA-A frequency with the infection rate and the infection rate with the severity rate showed a positive linear correlation. As a result, the weak HLA class I frequency correlated an exponential approximation increase with the severity rate.

Limitations

The limitations of this study are: 1) Data that only look at infection frequency and mortality until May 14, 2020, which is relatively early in the COVID-19 epidemic; 2) No statistical consideration given as to the effects of national economic, social, cultural, environmental factors, and medical level on the infection rate and severity in each country; 3) In a multi-ethnic country, only the representative racial group is examined, and racial weight is not considered; 4) No consideration given to biological risk factors other than HLA; 5) The biological mechanism of HLA against SARS-CoV-2 virus is not yet clear fully.

Since the data are from the early epidemic before the treatment method for COVID-19 was established, the effect of the treatment method is considered to be small. The other limitations are very complex and difficult to consider for each role that affects the disease. Regardless of these limitations, the statistical significance of HLA class I susceptible alleles against COVID-19 in this study is that HLA is a major factor for the infection and severity rate of the disease.

## Conclusions

When the number of binding repertoires in the HLA-A allele is reduced, it is considered that the cytotoxic T cell activity is also reduced. Weak HLA-A alleles with a low number of binding repertoires of SARS-CoV-2 peptides had an exponential correlation with the severity rate. The reason for the exponential approximation was that the weak HLA-A frequency showed a positive linear correlation with the infection rate as well as the infection rate with the severity rate. Weak alleles with a low number of binding repertoires in HLA-B alone do not correlate with the severity rate at all, but by forming a haplotype with weak HLA-A, the severity rate is stronger than that of weak HLA-A alone.
